# Longitudinal Measurements of Cerebrospinal Fluid Biomarkers in Parkinson's Disease

**DOI:** 10.1002/mds.26578

**Published:** 2016-02-16

**Authors:** Sara Hall, Yulia Surova, Annika Öhrfelt, the Swedish BioFINDER Study, Kaj Blennow, Henrik Zetterberg, Oskar Hansson

**Affiliations:** ^1^ Department of NeurologySkåne University HospitalMalmöSweden; ^2^ Department of Clinical SciencesLund UniversityMalmöSweden; ^3^ Department of Psychiatry and NeurochemistryInstitute of Neuroscience and Physiology, the Sahlgrenska Academy at the University of GothenburgGothenburg and MölndalSweden; ^4^ University College London Institute of NeurologyLondonUnited Kingdom; ^5^ Memory ClinicSkåne University HospitalMalmöSweden

**Keywords:** Parkinson's disease, cerebrospinal fluid, longitudinal, biomarkers, prognosis

## Abstract

**Objective:**

The purpose of this study was to investigate whether cerebrospinal fluid (CSF) levels of tau, phosphorylated tau, β‐amyloid_42_, α‐synuclein, neurofilament light, and YKL‐40 change over time and if changes correlate with motor progression and/or cognitive decline in patients with PD and controls.

**Methods:**

We included 63 patients with PD (nondemented) and 21 neurologically healthy controls from the prospective and longitudinal Swedish BioFINDER study, all of whom had clinical assessments and lumbar punctures at baseline and after 2 years.

**Results:**

CSF tau levels correlated strongly with α‐synuclein. The levels of CSF α‐synuclein, tau, phosphorylated tau, neurofilament light, and YKL‐40, but not β‐amyloid_42_, increased in CSF over 2 years in PD. No changes were seen in the control group. Studying patients with a short disease duration ( ≤ 5 years) and patients with a long disease duration ( > 5 years) separately, α‐synuclein and tau only increased in the PD group with long disease duration. In the PD group, an increase in phosphorylated tau over 2 years correlated with faster motor progression and faster cognitive decline. An increase in YKL‐40 over 2 years correlated with faster cognitive decline.

**Conclusion:**

CSF biomarkers reflecting Lewy body pathology and neurodegeneration (α‐synuclein), neuronal degeneration (tau, phosphorylated tau, and neurofilament light), and inflammation (YKL‐40) increase significantly over 2 years in PD. CSF levels of α‐synuclein and tau correlate and remain stable in the early symptomatic phase of PD but increase in the later phase. We hypothesize that CSF α‐synuclein levels might increase as a result of more intense neurodegeneration in PD with long disease duration. © 2016 The Authors. Movement Disorders published by Wiley Periodicals, Inc. on behalf of International Parkinson and Movement Disorder Society

It is important to understand the temporal changes in biomarkers to improve early diagnosis in PD, but also to identify biomarkers that could be used in therapeutic trials to evaluate new disease‐modifying therapies. Still, few longitudinal cerebrospinal fluid (CSF) biomarker studies have yet been performed. Many cross‐sectional studies during the past years have evaluated the diagnostic potential of α‐synuclein (α‐syn), neurofilament light (NFL), tau, phosphorylated tau (P‐tau), and amyloid β (Aβ) for PD‐related disorders. CSF levels of α‐syn have, when compared with controls, been found to be decreased in patients with PD as well as in patients with other synucleinopathies, for example, dementia with Lewy bodies and multiple system atrophy.^1^, ^2^, ^3^, ^4^, ^5^ On the other hand, CSF α‐syn shows a very marked increase in disorders with rapid neurodegeneration such as Creutzfeldt‐Jakob disease^6^ and also a clear increase in Alzheimer's disease (AD) when compared with both controls and patients with PD,^5^ indicating that α‐syn levels in CSF reflect neuronal degeneration. Levels of CSF biomarkers more commonly associated with AD, that is, tau, P‐tau, and Aβ_42_, have been found to be normal or slightly decreased in PD when compared with controls.^2^, ^3^, ^5^ In AD, tau and P‐tau have been shown to be increased when compared with controls, whereas Aβ_42_ has been shown to be decreased when compared with controls.^7^, ^8^ In patients with PD with dementia, most studies have shown normal levels of tau and P‐tau when compared with controls and normal or decreased levels of Aβ_42_.^5^, ^9^, ^10^, ^11^, ^12^, ^13^ We and another group have previously shown positive correlations between CSF α‐syn and tau as well as P‐tau.^3^, ^14^ However, this contradicts the results from Parnetti and colleagues,^15^, ^16^ who found negative correlations between α‐syn and tau. NFL (a biomarker for degeneration or injury of large myelinated axons) has been found to be increased in atypical parkinsonism when compared with PD.^5^, ^17^, ^18^ Furthermore, we have previously shown that NFL correlates with disease stage in PD.^5^ YKL‐40, also called chitinase‐3‐like 1 is a glycoprotein that is up‐regulated in inflammatory conditions.^19^ In the brain, YKL‐40 is expressed primarily in microglia and astrocytes.^20^, ^21^

Some longitudinal studies have investigated the association between baseline CSF biomarkers and subsequent disease progression in PD. Lower CSF Aβ_42_ has been shown to predict the subsequent development of cognitive decline in nondemented PD patients.^14^, ^16^, ^22^, ^23^, ^24^ Although several studies have shown decreased levels of CSF α‐syn in patients with PD,^1^, ^2^, ^3^, ^4^, ^5^ our group has shown that increased baseline levels of CSF α‐syn within the PD group predict future progression of motor symptoms and cognitive decline.^14^ This confirmed previous results showing that increased baseline levels of α‐syn are associated with future decline in cognitive function in PD patients.^25^ These results indicate that the association between CSF α‐synuclein and PD is complex, and we have hypothesized that CSF α‐synuclein starts to decrease during the preclinical stages of PD as a result of intraneuronal accumulation, but CSF α‐syn levels might also reflect neuronal damage with increasing values as the disease progresses. However, there are yet few studies that have assessed the longitudinal changes in CSF biomarkers over time in PD. Zhang and colleagues found that the rate of change in tau and tau/Aβ_42_ positively correlated with faster motor progression measured by UPDRS score, and Stewart and colleagues found a reduction in CSF α‐syn over a follow‐up of approximately 2 years, but this change did not correlate with worsening of motor symptoms or cognitive decline.^25^, ^26^

In the present prospective and longitudinal study, we investigated the CSF levels of tau, P‐tau, Aβ_42_, NFL, α‐syn, and YKL‐40 both at baseline and after 2 years of follow‐up in neurologically healthy elderly controls and patients with PD. Our aim was to investigate if there are changes over time in these CSF biomarker levels. We also studied if any changes in biomarker levels over time correlated with faster motor progression and/or cognitive decline in patients with PD.

## Methods

### Participants

This prospective and longitudinal study was performed at the Clinic of Neurology, Skåne University Hospital, Sweden, as part of the Swedish BioFINDER Study (www.biofinder.se). The study participants are primarily recruited from the southern region of Sweden and are followed with repeated neurologic, psychiatric, and cognitive assessments and collection of CSF and blood samples under standardized conditions. In this study, we included 63 patients with PD (nondemented), of whom 37 had short disease duration ( ≤ 5 years disease duration) and 26 had long disease duration ( > 5 years disease duration) who had had lumbar puncture at baseline and at the 2 year follow‐up. Of the patients with short disease duration, 11 were De Novo. The patients with PD met the National Institute of Neurological Disorders and Stroke Diagnostic Criteria for PD.^27^ None of the PD patients met criteria for PD with dementia at baseline.^28^ We also included 21 neurologically healthy controls with lumbar puncture at baseline and at the 2 year follow‐up. All control participants underwent cognitive testing and neurologic examination by a medical doctor, and individuals with objective cognitive or parkinsonian symptoms were not included as controls in the present study.

At baseline and at the 2 year follow‐up, a thorough medical history was taken and the patients underwent extensive testing for both motor symptoms and cognition. The patients were examined by a physician experienced in movement disorders and a registered research nurse using the UPDRS III, the Hoehn and Yahr Scale, and the Timed Up and Go test (TUG) among other scales.^29^, ^30^, ^31^ The study participants' cognitive function was assessed using the Mini Mental State Examination (MMSE), the Alzheimer's Disease Assessment Scale (ADAS) items 1 to 3, and the 1‐minute phonetic verbal fluency test or letter fluency test.^32^, ^33^, ^34^

All individuals gave written informed consent. The study procedure was approved by the local ethics committee at Lund University Sweden and conducted according to the Helsinki Declaration.

### CSF Samples

CSF samples were obtained by lumbar puncture in the L3/L4 or L4/L5 interspace with patients nonfasting. The samples were collected in polypropylene tubes and gently mixed to avoid gradient effects. All samples were centrifuged within 30 minutes at + 4°C at 2000*g* for 10 minutes to remove cells and debris and then stored in aliquots at −80°C pending biochemical analysis.

CSF Aβ_42_, tau, and P‐tau were analyzed using AlzBio3 (Fujirebio, Ghent, Belgium), NFL was analysed using the NF‐light assay (UmanDiagnostics, Umeå, Sweden), YKL‐40 was analyzed using the Human Chitinase 3‐like 1 Quantikine ELISA Kit (R&D, Minneapolis, Minnesota), α‐syn was analyzed using the Covance assay (Covance, Dedham, Massachusetts), and haemoglobin was analyzed with an assay provided by Bethyl Lab, Inc. (Montgomery, Texas). For α‐syn, only samples with haemoglobin <200 ng/ml were used; consequently, 4 samples from PD patients (all PD with long disease duration) and 1 sample from the controls were excluded, all baseline samples.

For P‐tau there were 8 missing in the control group and 16 missing in the PD group. Baseline and follow‐up CSF samples were always analyzed in the same batch. All analyses were performed using 1 batch of reagents by board‐certified laboratory technicians who were blinded to clinical data. Intra‐assay coefficients of variation were below 10%.

### Statistics

The statistical analyses were accomplished with SPSS for Windows, version 22.0 (SPSS Inc., Chicago, Illinois). To compare demographic and CSF baseline data between groups, the Mann–Whitney *U* test was used for continuous variables. The Pearson chi‐squared test was used to compare proportions. Univariate associations between 2 continuous variables were analyzed using Pearson's r (normally distributed variables) or Spearman's rho (skewed variables and/or ordinal data). Correlations between CSF biomarkers were investigated with linear regression to correct for confounding factors. To investigate changes in CSF biomarker levels over time we used a paired *t* test, and in the case of nonnormally distributed data we used Wilcoxon signed‐rank test. Correlations between baseline data and changes over 2 years in CSF biomarker levels were investigated with bivariate correlations. Correlations between changes over 2 years in CSF biomarker levels and changes over 2 years in clinical test scores as well as correlations with changes in other CSF biomarkers were then investigated with a linear regression, adjusting for age and Levodopa equivalent (LED).

## Results

### Demographics

Demographics and clinical characteristics of study participants at baseline and at 2 years are given in Table 1. CSF biomarker levels at baseline and at 2 years are given in Table 2. There were no gender effects. In the PD group, age correlated positively with α‐syn (*P* = .028, *R* = .286), tau (*P* = .001, *R* = .394), YKL‐40 (*P* < .001, *R* = .576), and NFL (*P* < .001, *R* = .517) and negatively with Aβ_42_ (*P* = .037, R = −.263). In the control group, age correlated positively with YKL‐40 (*P* = .014, *R* = .525) and NFL (*P* = .002, *R* = .643) and negatively with Aβ_42_ (*P* = .023, *R* = −.495). The levels of NFL, but no other CSF biomarkers, correlated with disease duration (*P* = .027, *R* = −.279). None of the CSF biomarkers correlated with disease severity as measured by the Hoehn &Yahr or UPDRS III. In the PD group, LED correlated with Aβ_42_ (*P* = .017, *R* = .300), but not with any of the other CSF biomarkers. In the PD group as a whole and in the PD group with short disease duration, but not in the PD group with long disease duration, there was a significant worsening in the Hoehn & Yahr score (*z* = −2.486, *P* = .013 and *z* = −2.366, *P* = .018, respectively) and a significant increase in LED (*z* = −3.377, *P* < .001 and *z* = −4.405, *P* < .001, respectively) over 2 years. In the PD group with long disease duration, there was a significant worsening in letter fluency (*t*_24_ = 4.612, *P* < .001). There were no significant changes in clinical test scores in the control group over 2 years.

**Table 1:** Demographics

	Control (n = 21)	PD total (n = 63)	PD with short disease duration (n = 37)	PD with long disease duration (n = 26)
Age at baseline	65.7 (6.8)	64.7 (9.4)	65.4 (10)	63.7 (8.6)
Women/Men	13/8	21/42^b^	14/23	7/19^b^
Disease duration at baseline	NA	5.5 (4.0)	2.7 (1.4)	9.6 (2.9)^c^
Hoehn & Yahr score at baseline	NA	2.0 (0.7)	1.9 (0.7)	2.2 (0.5)
Hoehn & Yahr score at 2 years	NA	2.3 (0.7)	2.3 (0.7)	2.3 (0.7)
UPDRS III score at baseline	1.5 (2.2)	19.2 (9.4)^a^	17.3 (7.6)^a^	22.0 (11.0)^a^
UPDRS III score at 2 years	2.2 (2.6)	17.7 (11.7)^a^	15.7 (11.1)	20.7 (12.2)
MMSE at baseline	28.1 (1.8)	28.5 (1.4)	28.6 (1.3)	28.2 (1.5)
MMSE at 2 years	28.4 (1.5)	28.0 (2.2)	28.1 (1.9)	27.8 (2.6)
Letter S fluency at baseline	16.7 (5.0)	15.5 (5.3)	14.3 (4.2)	17.2 (6.3)^d^
Letter S fluency at 2 years	16.0 (5.9)	14.4 (6.5)	14.6 (6.2)	14.1 (7.0)
Levodopa equivalent at baseline	NA	565 (469)	279 (248)	972 (404)^c^
Levodopa equivalent at 2 years	NA	767 (389)	499 (212)	939 (443)^c^

The data are presented as mean (standard deviation). NA, not applicable.

Letter fluency was missing in 1 PD patient (with long disease duration).

a *P* < .001 versus controls.

b *P* < .05 versus controls.

c *P* < .001 versus PD with short disease duration.

d *P* < .05 versus PD with short disease duration.

**Table 2:** CSF biomarkers at baseline and at 2 years

	Control (n = 21)	PD total (n = 63)	PD with short disease duration (n = 37)	PD with long disease duration (n = 26)
α‐Syn at baseline	1975 (758) (1127–4346)	1763 (496) (706–2857)	1718 (479) (1030–2857)	1839 (527) (706–2644)
α‐Syn at 2 years	1864 (623) (1119–3643)	1830 (569) (773–3070)	1746 (466) (1141–2986)	1949 (681) (773–3070)
Aβ42 at baseline	410 (197) (177–1086)	415 (120) (187–838)	406 (122) (192–838)	428 (118) (187–646)
Aβ42 at 2 years	410 (152) (156–664)	434 (153) (144–761)	420 (137) (155–761)	454 (173) (144–760)
Tau at baseline	100 (43) (43–214)	81 (31) (34–178)	82 (35) (34–178)	81 (24) (34–155)
Tau at 2 years	97 (40) (39–185)	86 (34) (35–203)	85 (36) (38–203)	88 (32) (35–181)
P‐tau at baseline	50 (18) (29–83)	40 (11) (21–65)	40 (11) (21–65)	40 (10) (23–61)
P‐tau at 2 years	58 (25) (27–110)	44 (14) (10–87)	44 (11) (27–76)	45 (17) (10–87)
NFL at baseline	852 (457) (443–2532)	913 (569) (4372–4328)	959 (637) (440–4328)	846 (461) (372–2295)
NFL at 2 years	898 (467) (424–2174)	994 (424) (367–2715)	992 (327) (434–1677)	998 (541) (367–2715)
YKL‐40 at baseline	183,515 (60,371)	164,073 (57,190)	170,582 (60,739)	154,809 (51,448)
	(100,320–341,183)	(47,402–328,160)	(47,402–328,160)	(86,916–303,987)
YKL‐40 at 2 years	187,602 (61,295)	178,964 (68,955)	183,049 (72,223)	173,152 (64,967)
	(101,887–357,882)	(47,752–350,088)	(47,752–350,088)	(67,976–334,806)

The data are presented in ng/L as the mean (standard deviation) as well as range (minimum–maximum). For P‐tau analyses, 8 values were missing in the control group and 16 values were missing in the PD group (1 PD with short disease duration and 15 PD with long disease duration) as a result of technical errors. For α‐synuclein (α‐syn), only samples with Hb < 200 ng/ml were used, and consequently 4 samples from PD patients (all with long disease duration) and 1 sample from controls were excluded. Amyloid β, Aβ; P‐tau, phosphorylated tau; NFL, neurofilament light.

### Correlations Between CSF Biomarkers at Baseline

In the PD group and in the control group, α‐syn correlated with tau (*P* < .001, β > .84), Aβ_42_ (*P* < .05, β > .30), and YKL‐40 (*P* < .001, β > .78), when adjusting for age and also LED in the case of PD. Tau correlated with YKL‐40 (*P* < .03, β > .52) and NFL (*P* < .002, β > .39) when adjusting for age and also LED in the case of PD.

In the PD group but not in the control group, P‐tau correlated with tau (*P* = .001, β = .440), α‐syn (*P* < .001, β = .541), and YKL‐40 (*P* = .013, β = .421), adjusting for age and LED.

### Change Over 2 Years in Levels of CSF Biomarkers

There were no significant changes over time in CSF biomarkers in the control group.

**Figure 1:**
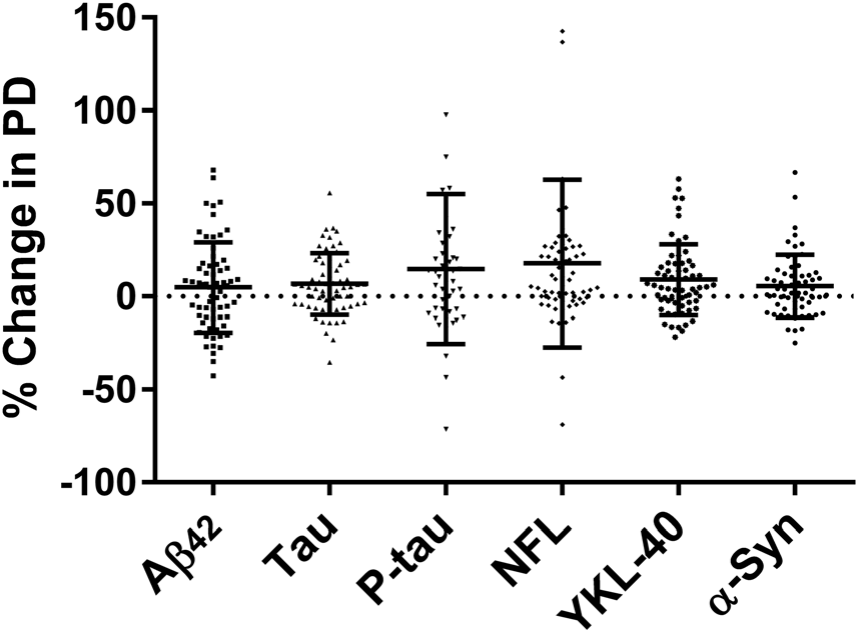
Percent change in CSF levels of amyloid β_42_ (Aβ_42_), α‐synuclein (α‐syn), tau, phosphorylated tau (P‐tau), neurofilament light (NFL), and YKL‐40 over 2 years. Scatter dots of change in percentages in CSF levels of Aβ_42_, tau, P‐tau, α‐syn, NFL, and YKL‐40 over 2 years. Lines represent means and standard deviations. One data point for P‐tau at 200% and 1 data point for NFL at 276% are outside the axis limit.

After 2 years of follow‐up, the PD group exhibited significantly increased levels of the CSF biomarkers tau (*t*_62_ = −2.570, *P* = .013), P‐tau (*t*_46_ = −2.458, *P* = .018), α‐syn (*t*_58_ = −2.350, *P* = .022), NFL (*z* = −3.769, *P* < .001), and YKL‐40 (*t*_62_ = −3.682, *P* < .001) when compared with baseline levels (Figure 1 and Supplementary Figure S1).

**Figure 2:**
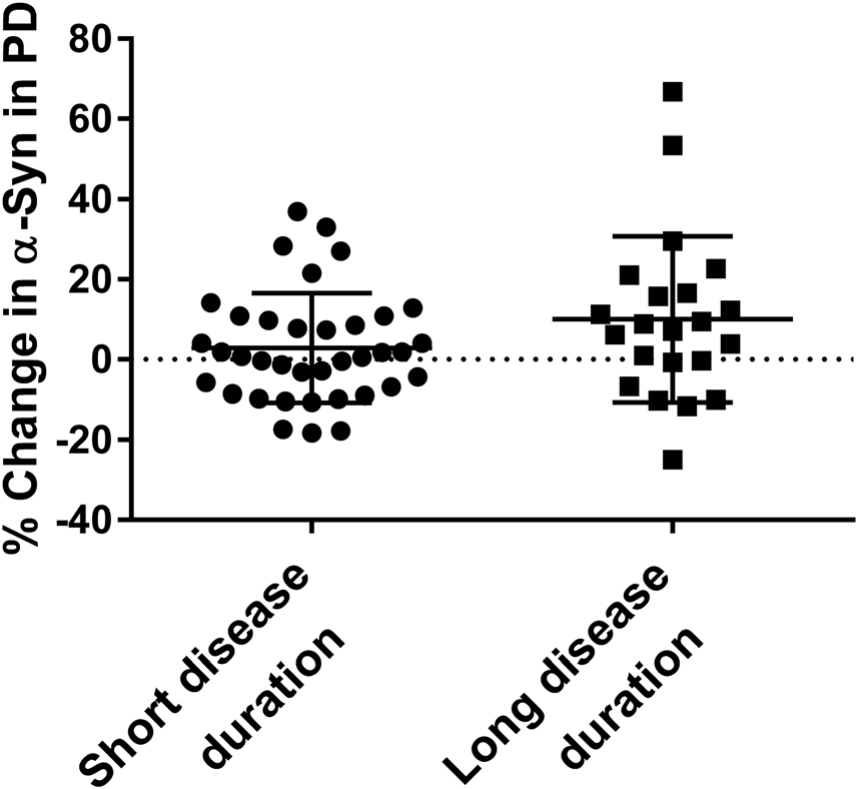
Percent change in CSF levels of α‐synuclein (α‐syn) in PD with short and long disease duration over 2 years. Levels of CSF α‐syn increase in the PD group with long disease duration over 2 years but not in the PD group with long disease duration. Scatter dots of change in percentages in CSF levels of α‐syn in PD with short and long disease durations. Lines represent means and standard deviations.

In the PD patients with short disease duration ( ≤ 5 years; n = 37), there were significantly increased levels of CSF biomarkers in NFL (*z* = −3.079, *P* = .002) and YKL‐40 (*t*_36_ = −2.675, *P* = .011) after 2 years. There was no significant increase in α‐syn or tau levels in the PD group with short disease duration (Figure 2 and Supplementary Figure S2). Furthermore, in the PD patients with mild and unilateral PD symptomatology (ie, Hoehn &Yahr score of < 2; n = 15) at baseline only NFL increased significantly over 2 years (*z* = −2.101, *P* = .036).

In the PD patients with long disease duration ( > 5 years; n = 26), there were significantly increased levels of CSF biomarkers in α‐syn (*z* = −2.192, *P* = .028), tau (*z* = −2.437, *P* = .015), NFL (*z* = −2.210, *P* = .027), and YKL‐40 (*z* = −2.222, *P* = .026) after 2 years (Figure 2 and Supplementary Figure S2). In PD patients with more severe and bilateral disease (ie, Hoehn &Yahr score of ≥ 2; n = 48) at baseline, levels of the same CSF biomarkers increased significantly over 2 years, that is, α‐syn, *t*_43_ = −2.232, *P* = .031; tau, *z* = −2.257, *P* = .024; P‐tau, *t*_35_ = −2.135, *P* = .040; NFL, *z* = −3.185, *P* = .001; and YKL‐40, *z* = −3.241, *P* = .001.

### Correlations Between Changes in CSF Biomarkers Over 2 Years in the PD Group

An increase in α‐syn levels correlated with increases in tau (*P* < .001, β = .583), P‐tau (*P* = .002, β = .470), and YKL‐40 (*P* < .001, β = .564) over a 2‐year period in PD. Furthermore, increases in tau levels correlated with increases in the levels of P‐tau (*P* < .001, β = .496), YKL‐40 (*P* = .001, β = .401), and NFL (*P* = .007, β = .347). Increases in P‐tau correlated with increases in YKL‐40 (*P* = .012, β = .375), all adjusted for age and LED (Supplementary Table S1).

### Correlations Between Baseline CSF Data and Changes in CSF Biomarkers Over 2 Years

Higher levels of P‐tau at baseline correlated with decreases in Aβ_42_ over 2 years (*P* = .001, *R* = −.454). None of the other CSF biomarkers at baseline predicted change in CSF biomarkers over 2 years.

### Correlations Between Changes in CSF Biomarkers and Changes in Clinical Test Scores Over 2 Years in the PD Group

**Figure 3:**
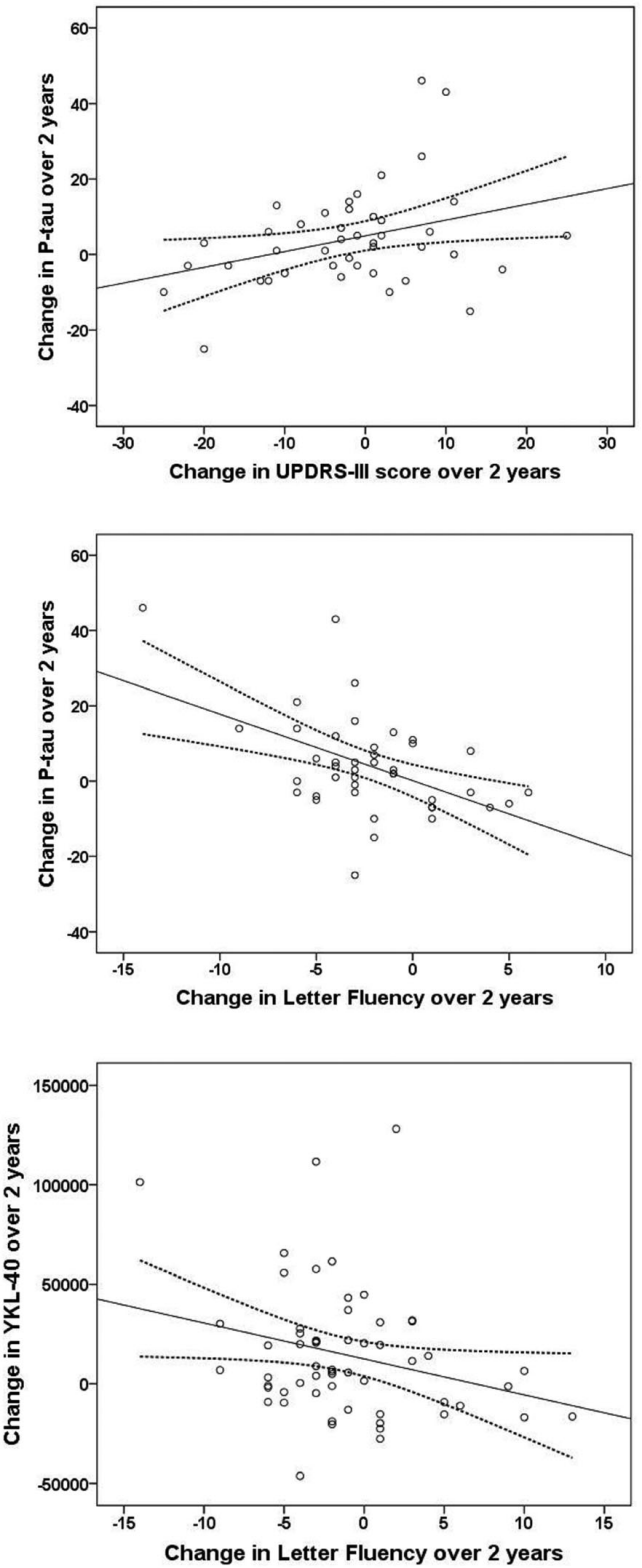
Correlations of changes CSF P‐tau with change in UPDRS III and letter fluency and CSF YKL‐40 with change in letter fluency over 2 years. Correlations of change in CSF levels of P‐tau with change in UPDRS III (A) and letter fluency (B) in the PD group. Correlations of change in CSF levels of YKL‐40 with change in letter fluency (C) in the PD group. Solid line indicates a linear regression, and dotted lines are 95% confidence intervals.

An increase in P‐tau over 2 years in the whole PD group correlated with worsening of motor symptoms over 2 years as measured by the UPDRS III (*P* = .048, β = .292), but not TUG, and with worsening of cognitive executive function as measured by letter fluency (*P* = .002, β = −.440), but not MMSE, when adjusting for age and LED. An increase in YKL‐40 over 2 years correlated with worsening of cognitive function as measured by letter fluency (*P* = .032, β = −.276), but not MMSE in PD when adjusting for age and LED (Figure 3). Changes in CSF levels of α‐syn, tau, and NFL over 2 years did not correlate with changes in motor symptoms (as measured by UPDRS III or TUG) or cognitive function (as measured by letter fluency or MMSE) when adjusting for age and LED.

## Discussion

In this prospective longitudinal study, we showed that levels of the CSF biomarkers tau, P‐tau, α‐syn, NFL, and YKL‐40 increase significantly over a period of 2 years selectively in the PD group, whereas control individuals had stable levels. Furthermore, we found that α‐syn and tau levels are stable early in the symptomatic disease process, but they increase over time later in the disease course and in more severe disease, with CSF levels of α‐syn and tau correlating with each other. We also found that a more rapid increase in CSF levels of P‐tau correlates with faster motor progression as well as cognitive decline as measured by the UPDRS III and letter fluency, respectively, and that a more rapid increase in CSF levels of YKL‐40 is associated with faster cognitive decline as measured by letter fluency.

Originally, CSF α‐syn tests were developed with the hope that they would reflect Lewy body pathology. Indeed, most synucleinopathies show slightly reduced CSF levels of α‐syn when compared with the levels seen in neurologically normal control individuals. However, this reduction is subtle when compared with the reduced CSF Aβ_42_ levels seen in cerebral β‐amyloidoses such as AD and dementia with Lewy bodies, and it is presently unclear exactly what CSF α‐syn concentrations represent. In the present study, CSF α‐syn levels correlated strongly with CSF tau, an established marker of neurodegeneration.^35^ Although levels of α‐syn are moderately decreased in synucleinopathies, emerging evidence suggests that increased CSF levels of the presynaptic protein α‐syn are associated with more intense neurodegeneration^2^, ^4^, ^5^, ^6^ as well as a worse prognostic outcome in PD.^14^, ^25^ However, it is still unclear why the levels of CSF α‐syn do not seem to correlate with disease duration in cross‐sectional analyses,^4^, ^14^, ^15^ but disease duration is a retrospective and not very precise measure when used on its own.

One study has previously found a reduction in CSF α‐syn during a follow‐up of approximately 2 years in PD with short disease duration.^25^ We found that both CSF α‐syn and tau mainly increase during the later stages of the disease as well as in the more severe phases of PD, when the neurodegeneration is probably more widespread. These results indicate that the association between CSF α‐syn and PD is complex not only reflecting the disease itself but also the disease stage. We therefore hypothesize that the CSF levels of α‐syn are bimodal over time and start to decrease early, probably in the preclinical stage of the disease process relating to intracellular accumulation. However, CSF α‐syn levels might also reflect neuronal damage with increasing values as the disease progresses, which is in concordance with previous studies showing that CSF α‐syn is increased in neurodegenerative disorders, including AD and Creutzfeldt‐Jakob disease.^2^, ^4^, ^5^, ^6^ This fact could help explain the variable association of CSF α‐syn levels with PD across studies.^1^

We found that the increase in the CSF levels of P‐tau is linked to not only more rapid progression regarding cognitive executive function as measured by letter fluency but also more rapid motor progression as measured by the UPDRS III. CSF P‐tau is believed to reflect tau pathology. In postmortem studies, the tau protein has been found in Lewy bodies.^36^ In genomewide associations studies the *MAPT* gene has been linked to PD, and risk alleles of *MAPT* have been associated with parkinsonism.^37^, ^38^ Furthermore, α‐syn in both mouse and human models has been shown to contribute to the phosphorylation of tau.^39^, ^40^ Several studies show slightly decreased levels of tau in PD when compared with controls.^2^, ^3^, ^5^ The rate of change of tau has been shown to be associated with motor progression.^26^ Thus, there is evidence that tau plays a role in the pathophysiology of PD. We have previously shown that P‐tau at baseline is associated with faster motor progression, and findings in the present study show that the rate of increase in P‐tau is associated with faster motor progression.^14^ We thus confirm the importance of tau pathology in PD and its role in disease progression. We hypothesize that a more aggressive disease, as one expects later on in the disease process, leads to increased levels of tau, a marker of neurodegeneration as well as an increased release of α‐syn, a protein localized in the presynaptic nerve terminal. This in turn could lead to a greater phosphorylation of tau, which in turn might aggravate the disease progression even further.

We have previously shown that NFL is increased in PD patients with a more advanced disease stage.^5^ NFL is a marker of the degeneration of large myelinated axons and is thus a marker for neurodegeneration. This is consistent with the result in the present study showing an increase in NFL over time in PD patients but not in controls.

Inflammation has also been implicated in the pathogenesis of PD.^41^ In this study, we show that YKL‐40 increases over time in PD (with short as well as long disease duration) and that this increase correlates with faster cognitive decline as measured by letter fluency. In the brain, YKL‐40 is expressed in astrocytes and microglia, both associated with inflammation.^20^, ^21^ YKL‐40 has been found to be expressed in both acute neurological disorders and chronic neurological disorders, including traumatic brain injury, stroke, multiple sclerosis, amyotrophic lateral sclerosis, and AD.^42^, ^43^ In one positron emission tomography study on PD patients, evidence of activated microglia was found in the pons, basal ganglia, and frontal and temporal cortical regions.^44^ Microglial activation has been shown to correlate positively with severity of motor symptoms.^45^ This is in line with the present data that shows that increasing levels of YKL‐40 are associated with a worsening of cognitive function in PD. YKL‐40 might consequently be a possible marker in therapeutic trials, especially if immunomodulating therapies were to be studied.

The increase in CSF levels of tau, P‐tau, α‐syn, NFL, and YKL‐40 were modest but significant. However, there was substantial variability in the longitudinal changes in the CSF levels of the biomarkers between different individual patients, which may reduce the clinical usefulness of these findings. Naturally, there are also many potential preanalytical confounders that might affect the measured CSF biomarkers, even though lumbar puncture was performed under standardized conditions. Therefore, the present results need to be replicated in a larger cohort. Another limitation might be that even though we have taken great care to follow the diagnostic criteria, a definite diagnosis in PD requires neuropathology. Furthermore, the patients were examined in the ON state, which may reduce the reliability of the UPDRS III as a measure of disease severity over time. Also, with all of the clinical rating scales used in this study (possibly with the exception of Hoehn & Yahr), the score is a snapshot in time. Finally, the follow‐up period of the present study was relatively short. PD is a disease that develops over many years and in that scope, 2 years is not long enough to draw any definite conclusions regarding long‐term changes in CSF biomarkers. Thus, studies with a longer follow‐up time are needed, and we are currently collecting CSF biomarker data from 4 and 6 years of follow‐up. Also, to give further insight in the role of α‐syn in the pathophysiology of PD, it would be important in future studies to study changes in oligomeric, phosphorylated, and truncated forms of α‐syn as well as total α‐syn.

In conclusion, CSF biomarkers reflecting Lewy body pathology (α‐syn), neuronal degeneration (tau, P‐tau, NFL), and inflammation (YKL‐40) increase moderately, but significantly, over 2 years in PD. CSF levels of α‐syn and tau remain stable in the early symptomatic phase of PD but increase in the later phase. We hypothesize that CSF α‐syn levels might be a reflection of a more intense neurodegeneration in PD with long disease duration. To our knowledge, these new findings may in the future be of use in both group stratifications as well as monitoring in clinical trials. However, the results need to be validated in future studies.

## Author Roles

(1) Research Project: A. Conception, B. Organization, C. Execution; (2) Statistical Analysis: A. Design, B. Execution, C. Review and Critique; (3) Manuscript: A. Writing of the First Draft, B. Review and Critique.

S.H.: 1B, 1C, 2A, 2B, 3A

Y.S.: 1C, 2C, 3B

A.O.: 1C, 2C, 3B

K.B.:1B, 2B, 2C, 3B

H.Z.: 1B, 2B, 2C, 3B

O.H.: 1A, 1B, 2A, 2C, 3B

## Relevant conflicts of interest/financial disclosures

S.H., Y.S., A.Ö and O.H. report no disclosures relevant to the manuscript. H.Z. and K.B. are cofounders of Brain Biomarker Solutions in Gothenburg AB, a Gothenburg University Holding‐based platform company at the University of Gothenburg. K.B. has served on advisory boards for Immuno‐Biological Laboratories International and Roche Diagnostics.

## Supporting information

Additional Supporting Information may be found in the online version of this article at the publisher's web‐site.

Supplementary Information
