# Supplementary material for: Longitudinal Measurements of Cerebrospinal Fluid Biomarkers in Parkinson's Disease
Source: Mov Disord. 2016 Feb 16;31(6):898–905. doi: 10.1002/mds.26578 (PMC5067556; doi:10.1002/mds.26578)
Supplement: Supplementary file 1 — Supplementary Information [file MDS-31-898-s001.docx]

**Supplementary Material:**

**Longitudinal measurements of cerebrospinal fluid biomarkers in Parkinson’s disease**

Hall S MD, Surova Y MD, Öhrfelt A PhD, the Swedish BioFINDER study, Blennow K MD PhD, Zetterberg H MD PhD, Hansson O MD PhD

- Supplementary Table S1
- Supplementary Figure S1
- Supplementary Figure S2

**Supplementary Table S1**

**Correlations between changes in CSF biomarkers over 2 years in the PD group**.

|  | **Aβ_42_** | **Tau** | **P-tau** | **α-syn** | **NFL** | **YKL-40** |
| --- | --- | --- | --- | --- | --- | --- |
| **Aβ_42_** |  |  | p=0.004 r=0.408 |  |  |  |
| **Tau** |  |  | p<0.001 r=0.487 | p<0.001 r=0.588 | p=0.003 r=0.371 | p=0.002 r=0.382 |
| **P-tau** | p=0.004 r=0.408 | p<0.001 r=0.487 |  | p=0.005 r=0.424 |  | p=0.008 r=0.381 |
| **α-syn** |  | p<0.001 r=0.588 | p=0.005 r=0.424 |  |  | p<0.001 r=0.521 |
| **NFL** |  | p=0.003 r=0.371 |  |  |  |  |
| **YKL-40** |  | p=0.002 r=0.382 | p=0.008 r=0.381 | p<0.001 r=0.521 |  |  |

For α- syn, only samples with Hb < 200 ng/ml was used and consequently 4 samples from PD patients were excluded.

Statistical analysis was done using Pearson’s correlations

**
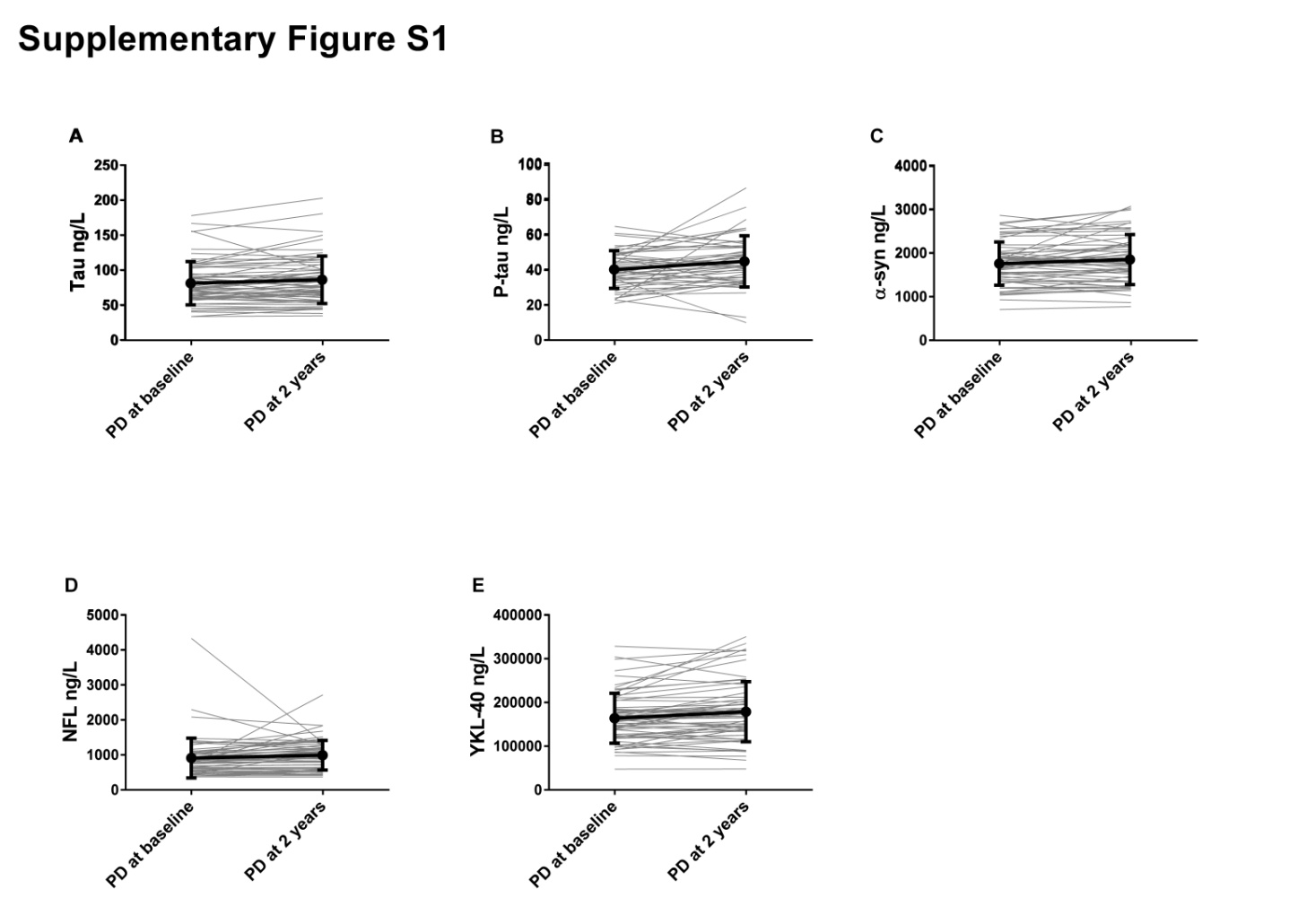
**

Multiple line graph of change in CSF levels of a) tau, b) P-tau, c) α-syn, d) NFL and e) YKL-40 over 2 years. Gray lines show change for the individual study participant. Solid lines represent mean and SD.


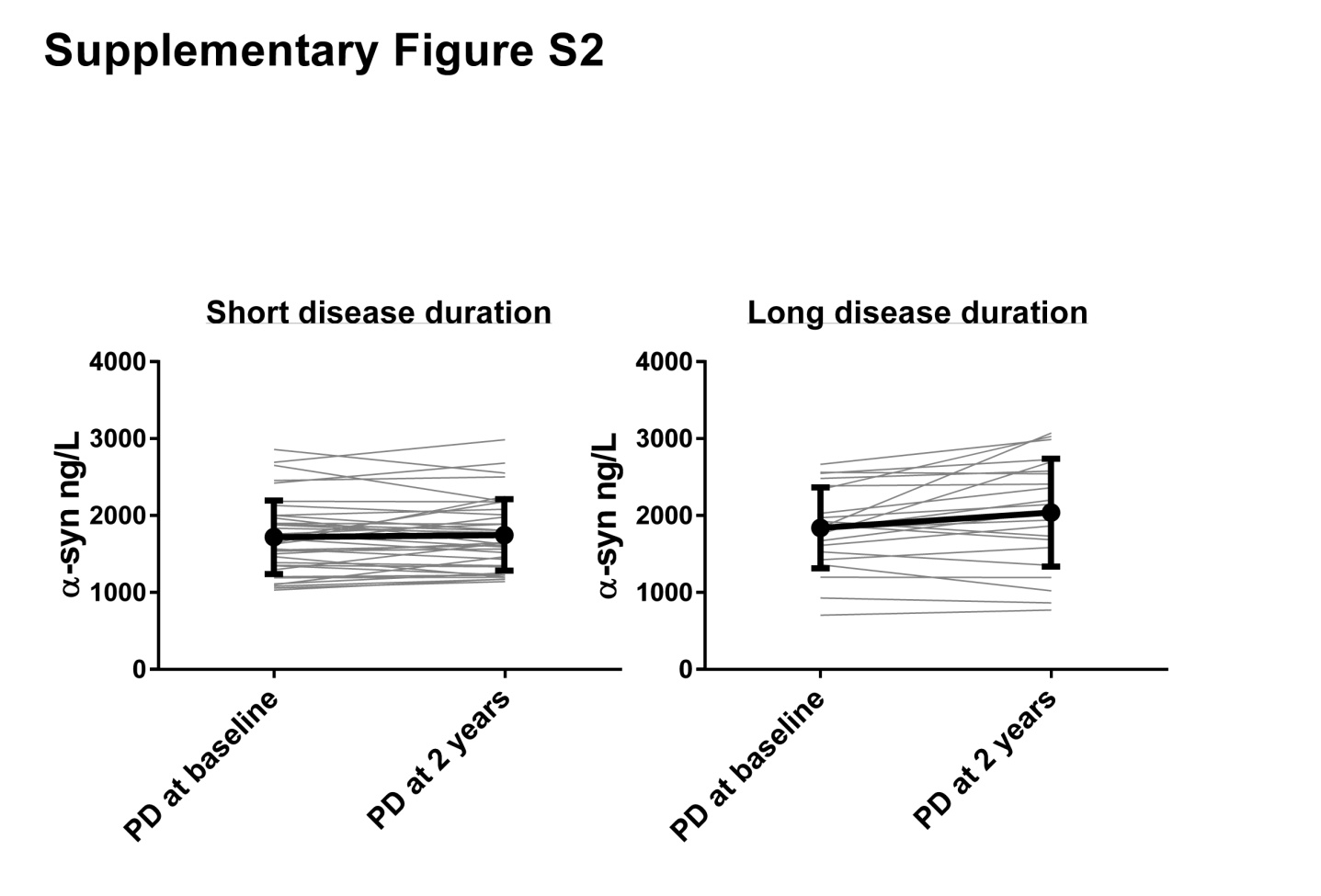


Multiple line graph of change in CSF levels of α-syn over 2 years in PD with short and long disease duration. Gray lines show change for the individual study participant. Solid lines represent mean and SD.
